# The Inclusion of Chiropractic Care in the Healthy China Initiative 2030

**DOI:** 10.7759/cureus.43068

**Published:** 2023-08-07

**Authors:** Eric Chun-Pu Chu, Andy Fu Chieh Lin, Valerie Chu

**Affiliations:** 1 Research, Chiropractic Doctors’ Association of Hong Kong, Hong Kong, CHN; 2 Chiropractic and Physiotherapy Centre, New York Medical Group, Hong Kong, CHN

**Keywords:** preventive medicine, china chiropractic, chiropractor, chiropractic, healthy china

## Abstract

The Healthy China Initiative 2030 represents a major shift in China’s healthcare policies for health promotion and disease prevention. Chiropractic care, with its focus on musculoskeletal health and nonpharmacological treatment, can contribute to the goals of this initiative. However, its potential contribution is hampered by the lack of official recognition and regulation in mainland China, which restricts its general awareness and integration into healthcare systems, and potentially leads to untreated musculoskeletal disorders.

This research proposes the inclusion of chiropractic care in the Healthy China Initiative 2030 framework. It provides an overview of the goals of this initiative and the current state of chiropractic care in China. The alignment of chiropractic principles and practices with the aims of the Healthy China Initiative 2030 is also discussed. Policy recommendations for integrating chiropractic care into the healthcare system are proposed, which include the establishment of education standards, licensing protocols, and collaborative research initiatives. Potential challenges, including regulatory barriers, a lack of awareness, and research limitations are highlighted. We also present potential strategies to leverage opportunities for promoting chiropractic care, such as the rising demand for musculoskeletal care. This research provides the first focused discussion on the integration of chiropractic care into China’s evolving preventive healthcare landscape under the Healthy China Initiative 2030.

## Editorial

Introduction

The Healthy China Initiative 2030 is a significant health policy that was launched by the Chinese government in 2016, with the aim of improving overall health standards in the country by 2030 [1]. The initiative sets forth two primary goals: (1) to enhance the health literacy of Chinese citizens to above 20% (A1) in the Chinese population, and (2) to increase their average lifespan by three years [2]. The strategy to achieve these goals is two-pronged, as it focuses on the prevention and control of diseases (via policy advocacy for health promotion and the creation of a health-friendly environment) and encourages health-related science and technology innovation [3]. Specific actions include strengthening health legislation, promoting healthy lifestyles, optimizing health services, improving health security, establishing an advanced health culture, and implementing monitoring and evaluation systems [4]. This initiative represents a shift from China’s previous healthcare model, which was largely focused on disease treatment, to a more comprehensive and integrated approach that emphasizes health promotion and prevention [1].

Chiropractic care, a hands-on healthcare discipline that focuses on the neuromusculoskeletal system, has been widely recognized for its benefits, particularly in terms of disease prevention and curative therapy. Chiropractic treatment primarily comprises spinal manipulation and can help maintain proper alignment of the body’s musculoskeletal structures, thereby leading to significant pain relief in the muscles, joints, bones, and connective tissue [5]. Such treatment can be especially beneficial for individuals with chronic conditions such as back pain, neck pain, and headaches. As chiropractic treatment is noninvasive and drug-free, it is often used to prevent the onset of musculoskeletal problems, reduce the need for medications, and improve overall health and well-being [5]. Moreover, it plays a pivotal role in the management of specific patient populations such as athletes and elderly individuals by improving physical function, preventing injuries, and enhancing recovery [6]. Thus, chiropractic care, with its dual role in prevention and treatment, can be an integral component of a comprehensive healthcare system.

The purpose of this article is to propose the inclusion of the chiropractic profession in the Healthy China Initiative 2030 [1]. Chiropractic care in both preventive and curative health contexts can be instrumental in achieving the health goals outlined in this initiative [5]. This article represents the first attempt to specifically address the role and potential contributions of chiropractic care within the framework of the Healthy China Initiative 2030. In this research, we provide a comprehensive discussion on the current state and potential of chiropractic care in China, examine how the profession aligns with the goals of the Healthy China Initiative 2030, and propose strategies for its inclusion in the initiative [7]. We highlight the potential impact of chiropractic care on the overall health of the Chinese population and advocate its integration into the broader health policy landscape in China.

Understanding chiropractic care in China

The discipline of chiropractic care focuses on disorders of the musculoskeletal and nervous systems and their effects on general health [5]. While chiropractic practitioners most commonly treat neck and back pain, they can also manage a wide range of other complex conditions [8]. Chiropractic care primarily involves manual therapy for the spine, peripheral joints, and soft tissues [5]. The techniques used in chiropractic care include, but are not limited to, spinal manipulation, therapeutic exercises, nutritional counseling, and lifestyle modification strategies [5]. The chiropractic approach to healthcare is holistic, emphasizing the body’s inherent recuperative power for healing [9]. The chiropractic scope of practice in some regions involves joint injection and surgery, functional medicine and neurology, and management of emotional symptoms. Chiropractors often emphasize the importance of spinal health for overall well-being based on the premise that spinal misalignments can interfere with the nervous system and result in diminished health [10].

Chiropractic care has been increasingly recognized in Hong Kong for its evidence-based benefits for various health conditions [11]. Numerous studies have demonstrated that chiropractic treatment can be effective in managing musculoskeletal pain, especially low back pain. For instance, a systematic review by Coulter et al. found that chiropractic care leads to significant improvements in pain intensity and disability in patients with low back pain [12]. Additionally, a comprehensive review showed that spinal manipulative therapy is as effective as other commonly used interventions for chronic low back pain [13]. Furthermore, a study by Chu et al. revealed that the risk of adverse events from chiropractic treatment is extremely low, thus indicating the safety of this treatment modality [14]. Chiropractors in Hong Kong sometimes manage nonmusculoskeletal symptoms of spinal origin, such as cervicogenic angina, dyspnea, and visual dysfunction [15-18].

The state of chiropractic care in China

Chiropractors are not officially recognized as medical professionals in mainland China. Despite the lack of official recognition, some chiropractic clinics that largely serve expatriate communities and wealthier Chinese citizens have been established in major cities, such as Beijing, Shanghai, and Guangzhou. Many chiropractors work in private [19] or local hospitals; some chiropractors serve on national sports teams and manage sports injuries [6,20,21]. However, comprehensive and current data on the number of practitioners, clinics, and patients are not readily available because of the informal status of the profession. Furthermore, the lack of a formal regulatory framework has implications for the quality of care, as it is difficult to ensure that all practitioners have the necessary qualifications. In Hong Kong, however, chiropractors are registered by law under the Chiropractors Registration Ordinance 1993 [22] and can practice in China through the mainland and Hong Kong Closer Economic Partnership Arrangement [23].

Given the lack of official recognition and regulation of chiropractic care in mainland China, public awareness and understanding of this treatment modality are limited. Although many studies have been conducted in these regions [24-26], no comprehensive investigations have been performed to gauge public perception of chiropractic care in China. Any available information is largely anecdotal, suggesting that those who are aware of chiropractic care often perceive it as a form of wellness care for musculoskeletal issues; this is particularly evident among the more affluent citizens and expatriate communities in large cities such as Beijing, Shanghai, Chengdu, Shenzhen, and Guangzhou. However, the majority of the Chinese population, especially those residing outside major cities, may have little or no knowledge of the profession. This situation contrasts with places such as Hong Kong, where chiropractic care is a recognized and regulated health profession and public awareness is consequently higher [27].

How chiropractic care aligns with the goals of the Healthy China Initiative 2030

Chiropractic care is aligned with several goals of the Healthy China Initiative 2030 [1]. One of these key objectives is to promote the prevention and control of chronic diseases, including musculoskeletal disorders [5]. As chiropractic care comprises noninvasive and nonpharmacological management of musculoskeletal conditions, it can enhance the quality of life of many individuals [12]. Previous research has suggested that chiropractic care can decrease gabapentin prescriptions by 47% [28]. Moreover, the Healthy China Initiative 2030 encourages the integration of traditional Chinese medicine (TCM) with other health services. Although chiropractic care is not a form of TCM, both disciplines share many common principles, particularly the emphasis on holistic and patient-centered care and the body’s self-healing capabilities, thus suggesting a potential synergy if integrated appropriately [29]. Finally, chiropractic care prioritizes patient education and promotes self-management strategies; these features are aligned with the goal of the Healthy China Initiative 2030 of empowering individuals to take charge of their health [30]. Therefore, chiropractic care can potentially contribute to the goals of the initiative, provided that there is further recognition of the profession and the development of relevant regulations in China.

Policy recommendations for the inclusion of chiropractic care

Several policy recommendations can be considered for integrating chiropractic care into the healthcare system under the Healthy China Initiative 2030. First, the Chinese government should officially recognize chiropractic care as a healthcare profession. This would establish a regulatory framework for chiropractor education, licensing, and practice [31]. This official recognition can be achieved by working with international chiropractic organizations to ensure that the standards of education and practice align with global norms. Second, the government should promote research on the clinical efficacy and cost-effectiveness of chiropractic care in the Chinese context. This can involve collaborations between Chinese researchers and their international counterparts who are experienced in chiropractic research [6,32-35]. Third, there should be initiatives to educate the public and other health professionals about the role of chiropractic care in managing musculoskeletal conditions and promoting overall health [36]. Lastly, policy initiatives should explore ways to integrate chiropractic care with other health services, including TCM, to offer a holistic, patient-centered approach to health and wellness [37].

As part of the implementation strategy for integrating chiropractic care into the healthcare system under the Healthy China Initiative 2030, it is crucial to systematically develop chiropractic services and education programs. First, establishing chiropractic educational programs based on international standards would be a step toward ensuring the quality of practitioners [31]. Collaboration with established chiropractic institutions worldwide can help establish these programs. Second, the development of a licensing and regulatory body can help maintain standards of practice and ensure patient safety [38]. Third, integrating chiropractic services into existing health services, such as hospitals and community health centers, can make them more accessible to the public. In addition, chiropractic services can be included in health insurance schemes, thereby making care more affordable [27]. Lastly, promoting local case reports on chiropractic care within China can help tailor services to the unique needs and context of Chinese populations [39-42].

Potential challenges and solutions

The inclusion of chiropractic care in the Healthy China Initiative 2030 may face several challenges. Regulatory issues are a significant hurdle, as chiropractic care is not yet officially recognized as a healthcare profession in China [31]. To overcome this, the Chinese government should work with international chiropractic organizations to establish a regulatory framework for chiropractic education, licensing, and practice. Another challenge is the lack of awareness about chiropractic care among both the general public and healthcare professionals [30]. Educational initiatives can be implemented to increase knowledge of the benefits and safety of chiropractic care [30]. Similarly, potential resistance from other healthcare professionals can be mitigated through interdisciplinary collaboration and by highlighting the complementary role of chiropractic care in patient management [12]. Finally, research barriers can be addressed by promoting collaboration between Chinese researchers and their international counterparts and by securing funding for research on the clinical efficacy and cost-effectiveness of chiropractic care in the Chinese context.

Several opportunities can be leveraged for the successful inclusion of chiropractic care in the Healthy China Initiative 2030. There is a clear demand for effective and nonpharmacological management strategies such as chiropractic care in China due to the increasing burden of musculoskeletal conditions, particularly lower back and neck pain [27]. The growing interest in complementary and alternative medicine in China has also provided an opportunity to introduce chiropractic care as an additional treatment option for patients [27]. Existing collaborations with international chiropractic organizations should be leveraged to develop high-quality education and training programs, as well as establish a regulatory framework for chiropractic practice [31]. Furthermore, China’s extensive health infrastructure should be utilized to make chiropractic services widely available by integrating them into hospitals and community health centers. Finally, the government’s commitment to improving public health under the Healthy China Initiative 2030 provides a supportive environment for the integration of chiropractic care [43].

Conclusions

This article highlighted the potential of chiropractic care to contribute to the goals of the Healthy China Initiative 2030. We provided an overview of chiropractic principles and practices, discussed the current limited state of the profession in China, and examined the alignment between chiropractic care and the objectives of the Healthy China Initiative 2030. We proposed policy recommendations for integrating chiropractic care into China’s healthcare system. These included the establishment of educational standards, practice regulations, research collaborations, awareness campaigns, as well as integration with existing health services. While we acknowledged challenges such as regulatory barriers, lack of awareness, and research limitations, we also highlighted strategies for leveraging opportunities presented by the rising demand for musculoskeletal care in China. We argue that the official recognition and integration of chiropractic care can potentially complement other preventive, rehabilitative, and curative healthcare services in China. Further discourse and policy action in this direction can contribute to the goals of improving population health, promoting active aging, and enhancing the quality of life.
